# High abundance of *Ralstonia solanacearum* changed tomato rhizosphere microbiome and metabolome

**DOI:** 10.1186/s12870-020-02365-9

**Published:** 2020-04-15

**Authors:** Tao Wen, Mengli Zhao, Ting Liu, Qiwei Huang, Jun Yuan, Qirong Shen

**Affiliations:** Jiangsu Provincial Key Lab for Organic Solid Waste Utilization, Jiangsu Collaborative Innovation Center for Solid Organic Wastes, Educational Ministry Engineering Center of Resource-saving fertilizers, Nanjing Agricultural University, Nanjing, 210095 China

**Keywords:** Rhizosphere soil microbiome, Rhizosphere metabolome, *Ralstonia solanacearum*, Pathogen abundance, Co-occurrence network

## Abstract

**Background:**

Rhizosphere microbiome is dynamic and influenced by environment factors surrounded including pathogen invasion. We studied the effects of *Ralstonia solanacearum* pathogen abundance on rhizosphere microbiome and metabolome by using high throughput sequencing and GC-MS technology.

**Results:**

There is significant difference between two rhizosphere bacterial communities of higher or lower pathogen abundance, and this difference of microbiomes was significant even ignoring the existence of pathogen. Higher pathogen abundance decreased the *alpha* diversity of rhizosphere bacterial community as well as connections in co-occurrence networks. Several bacterial groups such as *Bacillus* and *Chitinophaga* were negatively related to the pathogen abundance. The GC-MS analysis revealed significantly different metabolomes in two groups of rhizosphere soils, i.e., the rhizosphere soil of lower harbored more sugars such as fructose, sucrose and melibiose than that in high pathogen abundance.

**Conclusions:**

The dissimilar metabolomes in two rhizosphere soils likely explained the difference of bacterial communities with Mantel test. *Bacillus* and *Chitinophaga* as well as sugar compounds negatively correlated with high abundance of pathogen indicated their potential biocontrol ability.

## Background

Microbiota associated with the host plant, especially roots, determine the infection of the soil-borne pathogen [29]. Assembling a self-serving rhizosphere microbiota is vital for both plant and pathogen. Plants recruit beneficial microbes to stimulate plant growth, elicit plant systemic defense, and antagonize pathogens [3, 32]. This kind of recruitment is realized by the release of specific compound, and by alteration of hormone expression levels in plant [4, 35]. For plant pathogens, recognition of host plant via root exudates is the first step of successful invasion [7, 12]; in addition, suppression of host defenses and acquisition of essential nutrients also master the invasion events [7, 15, 44]. The interactions between pathogens or between pathogen and other microbes may promote pathogenicity by substance exchange or effectors gene expression enhancement [16, 31]. Such studies indicated that there is a pathogen helper during the process of pathogenesis. Investigation of rhizosphere microbiome after pathogen invasion may give insights on interactions between the host plant and pathogen as well as relationship between pathogen and other microbes.

Soil microbial community is causally affected by abiotic factors such as physiochemical soil properties, pH, temperature, and soil moisture [13, 25]. Whereas, little attention is given to the effects of soil metabolome on soil microbiota, such as sugars, organic acids, and other low molecular weight compounds, which can serve as available nutrients for soil microbes and can be responsible for the interactions of plants with rhizosphere microbes [26]. Root exudates are the main source of rhizosphere soil low molecular weight molecules, and changes in the root exudates composition can affect the composition of rhizosphere soil microbial community [17, 26, 48]. The soil nutrient condition and health status of host plant can also shift the composition of rhizosphere microbiota by affecting root exudates [30].

Species invasion has a strong impact on the original ecosystem through competition of resources and niches with the indigenous microbes [43]. In rhizosphere soil, pathogen invasion can also affect the microbial community by indirectly influencing the host [38, 41], for example, Berendsen et al [4] showed that *Arabidopsis thaliana* specifically promotes three bacterial species in the rhizosphere upon foliar defense activation by the downy mildew pathogen *Hyaloperonospora arabidopsidis*. As a gram-negative phytopathogenic bacterium, *Ralstonia solanacearum* leads to the global bacterial wilt disease [51–53]. It showed the rapid capacity to spread to the aboveground after invasion of xylem vessels [54–56] and then excessively produce the extracellular polysaccharides to block the water flow [57, 58].

In this study, the rhizosphere soil samples were collected from a 7-year-monoculture greenhouse characterized by a high abundance of *R. solanacearum* with 100% disease incidence at the last of fruiting stage previously. The tomato plants together with the soils were sampled at the late developmental stage. Total pathogen density was detected by q-PCR, low molecular weight molecules of soil were determined by GC-MS technology, and high throughput sequencing was used to determine changes in the composition of the rhizosphere bacterial community. We set up this study to answer two questions: (1) does *R. solanacearum* invasion determine the composition of rhizosphere microbial community? (2) do low molecular weight molecules of the rhizosphere soil play a role in the changes of the composition of rhizosphere microbial community?

## Results

### The abundance of *Ralstonia solanacearum* in tomato rhizosphere

**Fig. 1:**
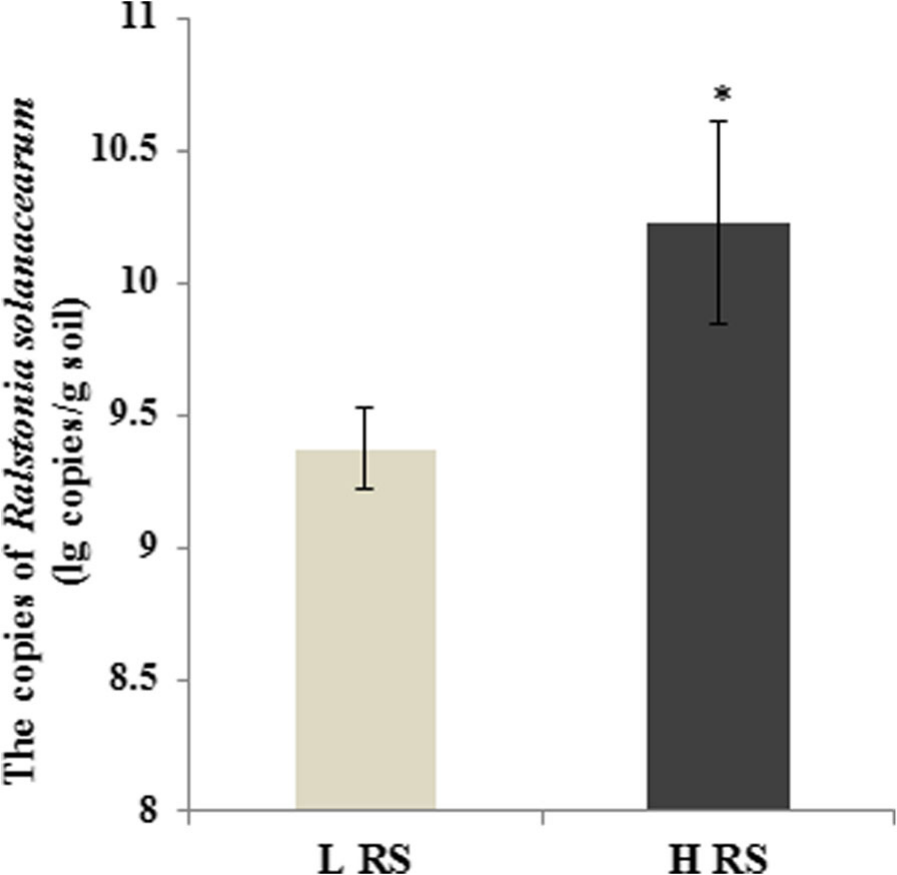
The abundance of RS in two rhizosphere soil samples measured by quantitative PCR

We selected 12 soil samples and divided them into higher RS abundance group (HRS) and lower RS abundance group (LRS), thus having 6 replicates of each group. The abundance of RS was significantly different (*p* < 0.05, t-test) between two groups (Fig. 1). Then 12 samples were sequenced with 16S rRNA amplicon by Illumina Hiseq technology to unfold the rhizosphere bacterial microbial community.

### Bacterial community composition

**Fig. 2:**
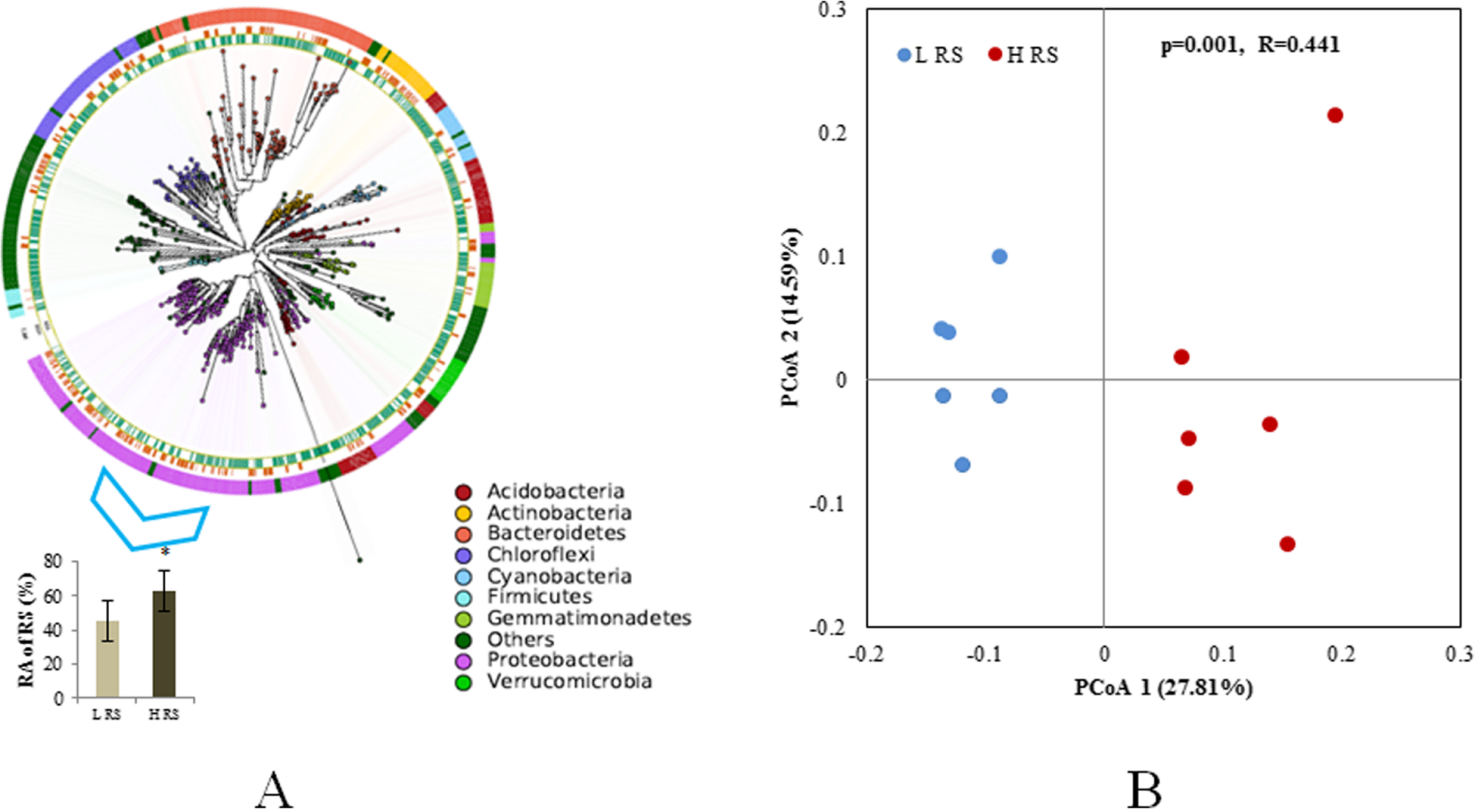
**a** The maximum-likelihood phylogenetic tree of all OTUs clustered. The outer ring shows the taxonomic group, the central ring shows the high abundance of RS group, and the inner ring shows the low abundance of RS group. The relative abundance of RS in two groups were present at the left bottom, and asterisk indicated the significant difference between two groups. **b** Principal coordinates analysis (PCoA) with Bray-Curtis dissimilarity of the rhizosphere bacterial community in low abundance of RS samples (LRS) and high abundance of RS samples (HRS)

The average read count of all samples was 15,143 (with the standard deviation (SD): 3416). The number of the OTUs could reflect the diversity of the bacterial communities and numbers of the OTUs range between 213 and 410 per sample with an average of 340 (SD: 59). The top four phylum with the highest relative abundance were *Proteobacteria* (76.72%), *Bacteroidetes* (13.63%), *Cyanobacteria* (4.36%), and *Actinobacteria* (1.98%) (Fig. 2a). There was a clear difference between two community composition evaluated with principal coordinates analysis (PCoA) and the difference of community composition were statistically significant through analysis of similarity (PERMANOVA) (*p* = 0.001, R2 = 0.44) (Fig. 2b). Upon closer inspection of these bacterial communities, one OTU belonging to *Ralstonia* was dominant among *Proteobacteria* (Fig. 2a). The sequencing data upon *Ralstonia* showed the similar result with the quantitative data that HRS group harbored a significant higher relative abundance than LRS groups (Fig. 2a). After the exclusion of *Ralstonia*, the differences among the two groups was still statistically significant (p = 0.001 in PERMANOVA, Supplementary Fig. 1).

**Fig. 3:**
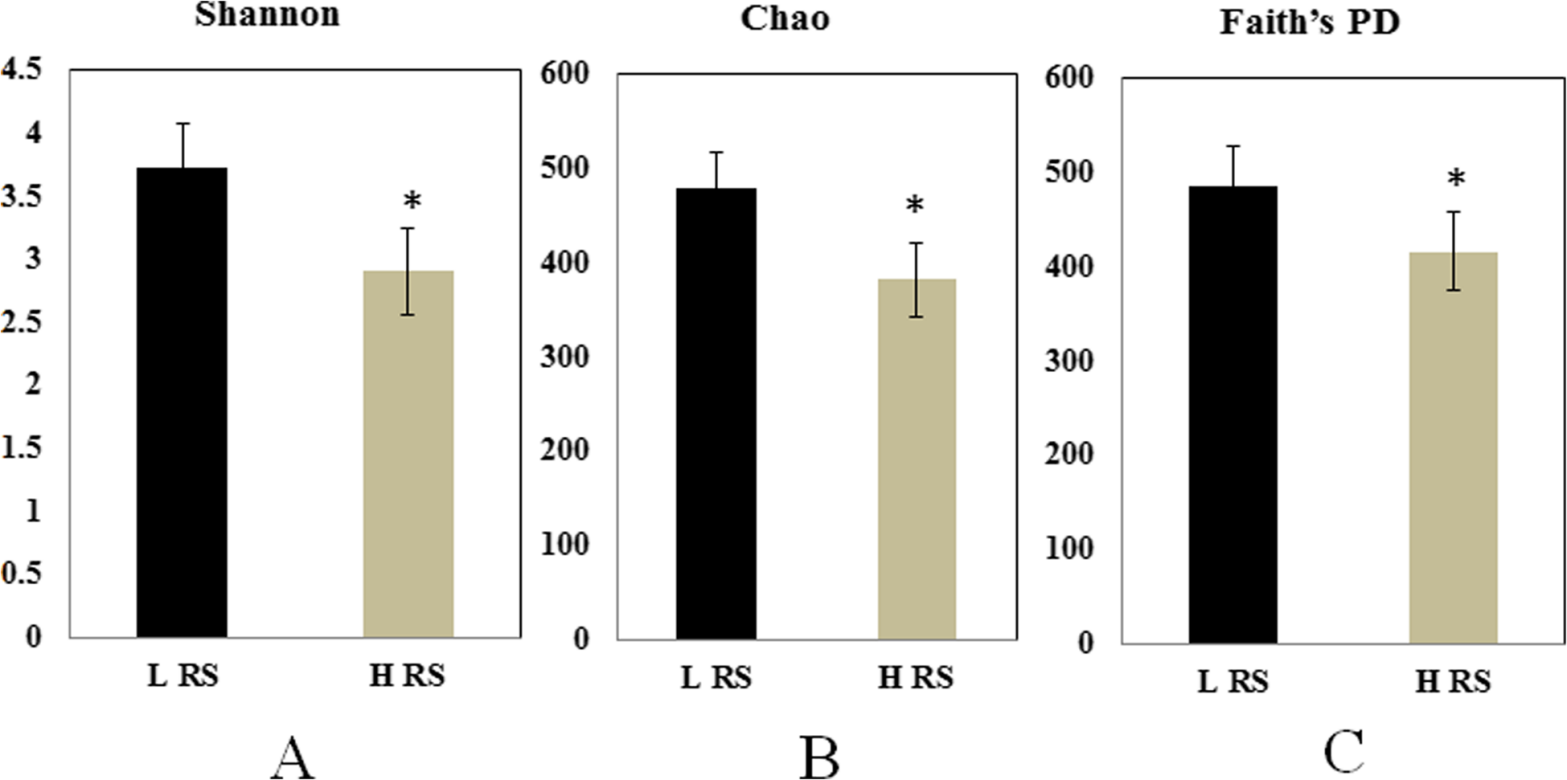
The *alpha* diversity indices of rhizosphere bacterial community in low abundance of RS (LRS) samples and high abundance of RS (HRS) samples. Shannon (**a**), Chao (**b**), and Faith’s PD (**c**). Asterisk indicated the significant difference between two groups

The *alpha* diversity indicators were calculated considering all the observed OTUs, the high abundance of *Ralstonia* decreased the *alpha* diversity of rhizosphere bacterial communities of HRS compared to LRS group (Fig. 3).

### Comparing changes in the rhizosphere bacterial co-occurrence networks

**Fig. 4:**
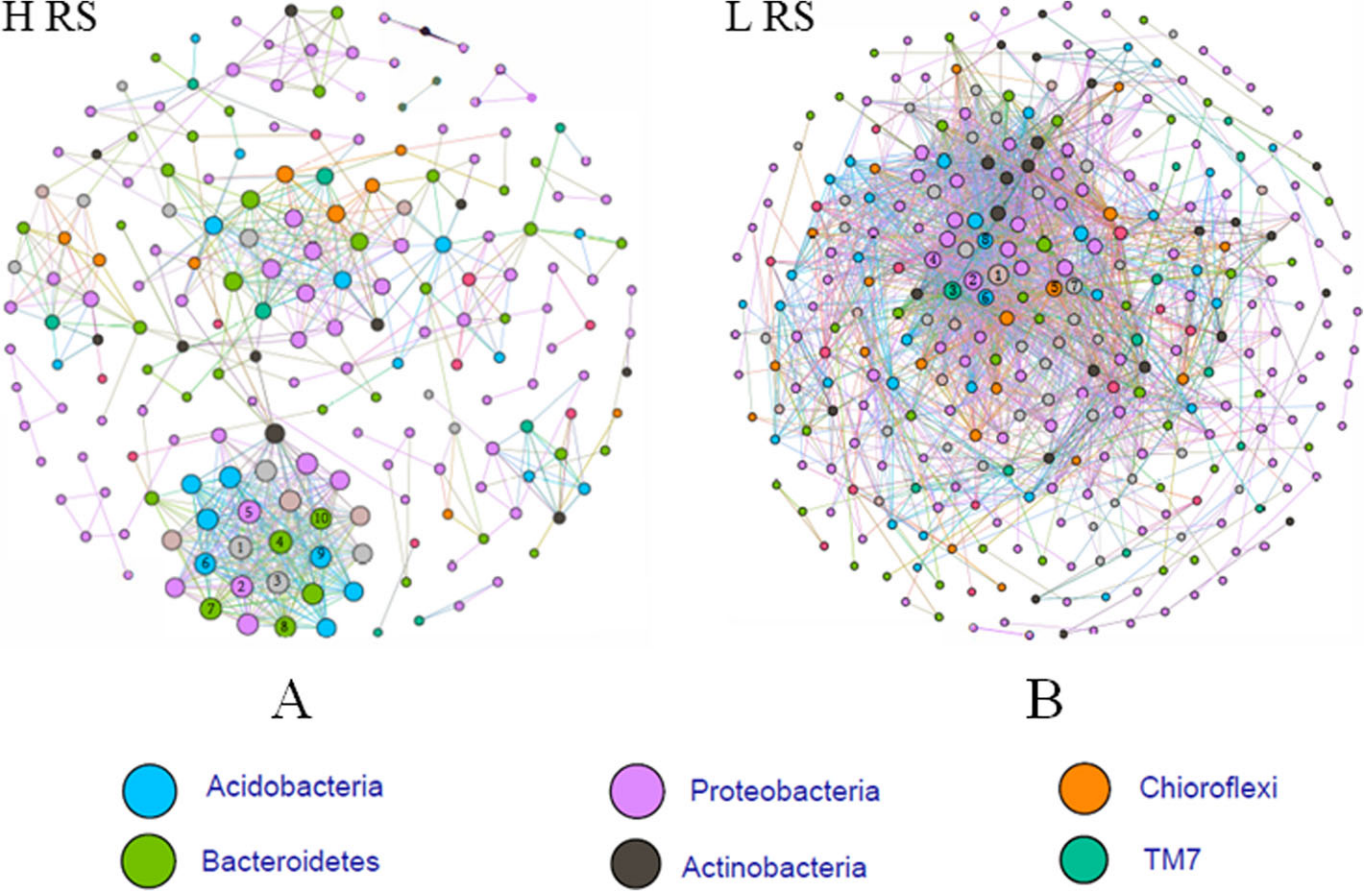
Co-occurrence network analysis of the rhizosphere bacterial community in high abundance of RS (HRS) samples (**a**) and low abundance of RS (LRS) samples (**b**). Co-occurrence networks of tomato rhizobacteria were constructed based on correlation analysis of taxonomic profiles. Connections are drawn between nodes that were significantly (*P* < 0.01; Spearman’s rank correlation test) and highly (Spearman’s r > 0.96) correlated. Hub bacteria for each network are ranked according to the number of connections in the network. For (A) 1): O_Solibacterales; 2): G_Flavisolibacter; 3): G_Paenibacillus; 4): F_Chitinophagaceae; 5): O_Sphingobacteriales; 6): O_MND1; 7): O_Solibacterales; 8): O_Sphingobacteriales; 9): G_GOUTA19; 10): F_Rhodospirillaceae. (B) 1): O_DS-18; 2): O_iii1–15; 3): Unassigned; 4): G_Candidatus Xiphinematobacter; 5): O_H39; 6): O_EW055; 7): O_MND1; 8): F_Xanthomonadaceae

To gain further insight into the effect of pathogen invasion on bacterial community, we constructed bacterial co-occurrence networks based on the correlation analysis of taxonomic profiles in high and low *Ralstonia* abundance rhizosphere of tomato (Fig. 4). The networks differed between two groups. In general, the higher abundance of *Ralstonia* led to a clear decrease in the number of connections. There were 197 and 310 nodes with 666 and 2153 links for HRS and LRS groups, respectively. The other global statistics of this network analysis are listed in Supplementary Table 1. Dominate hubs in two networks showed some differences, in detail, the major hub bacteria were *Paenibacillus*, *Flavisolibacter*, *Chitinophagaceae*, *Sphingobacteriales*, *MND1*, *Solibacterales*, *Rhodospirillaceae* and *GOUTA19* in the HRS rhizosphere, and *Xiphinematobacter*, *Xanthomonadaceae*, iii1–15, H39, EW055, MND1 and DS-18 in the LRS rhizosphere. The *Ralstonia* was connected with only one OTU classified as *Kaistobacter*, which may be potentially important bacterial group related to *Ralstonia* invasion.

### Potential bacterial taxonomies related to pathogen resistance

**Fig. 5:**
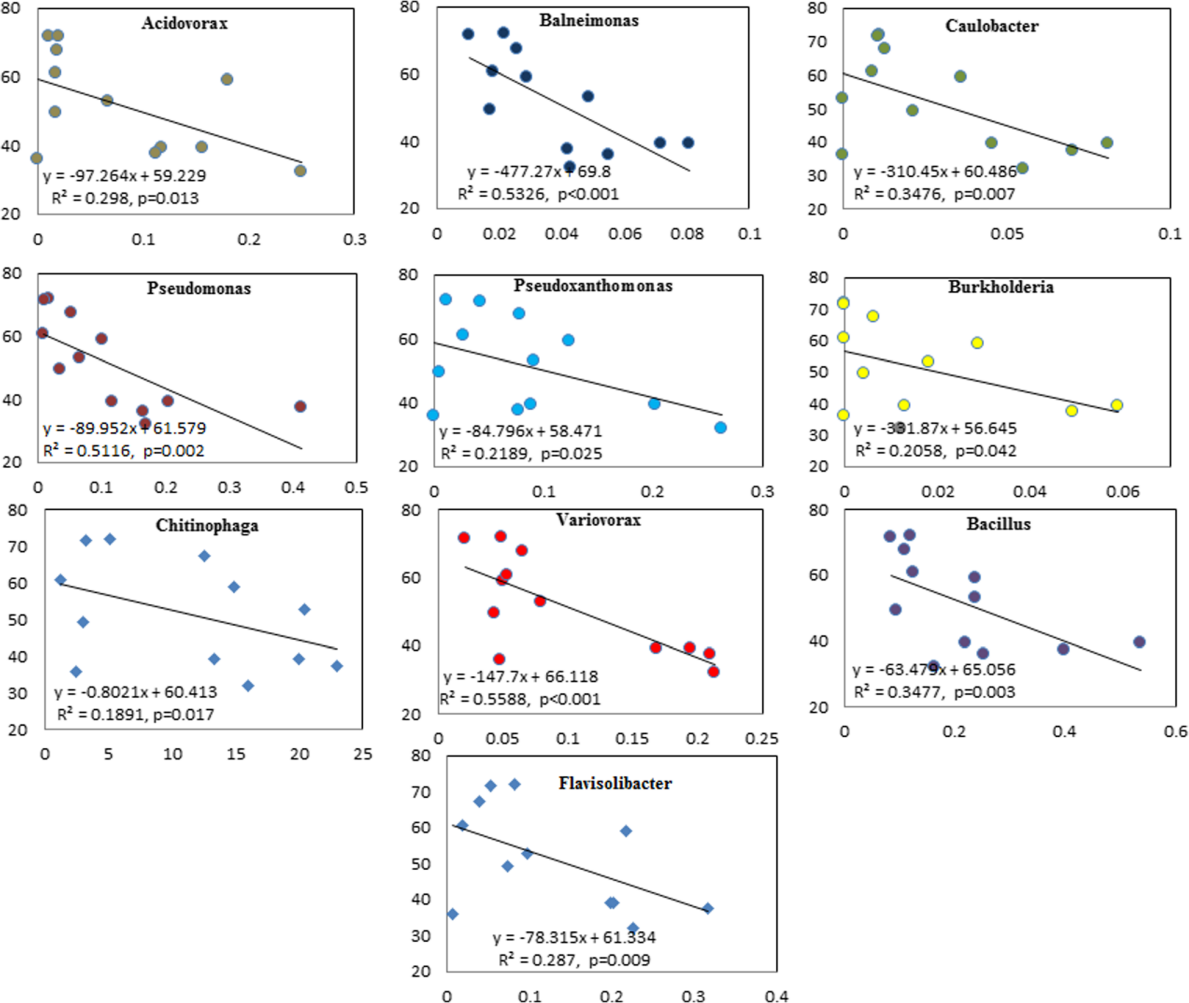
The relationship between targeted bacterial groups and RS in tomato rhizosphere bacterial microbiome. x-axis means the relative abundance of targeted bacterial groups (%), and y-axis means the relative abundance of RS (%)

To investigate the potential bacterial groups involved in pathogen multiplication, we further screened the relative abundance at the genus level. Ten genera appeared with their relative abundance negatively related to the pathogen abundance, and their relative abundance were all significantly higher in the LRS group than in HRS group (Fig. 5). For example, *Chitinophaga* was significantly (*p* < 0.05, t-test) higher in LRS group than in HRS group with the relative abundance of 17.95 and 4.6%, respectively.

### Characterization of rhizosphere soil metabolites profile and relationship with the composition of bacterial community

**Fig. 6:**
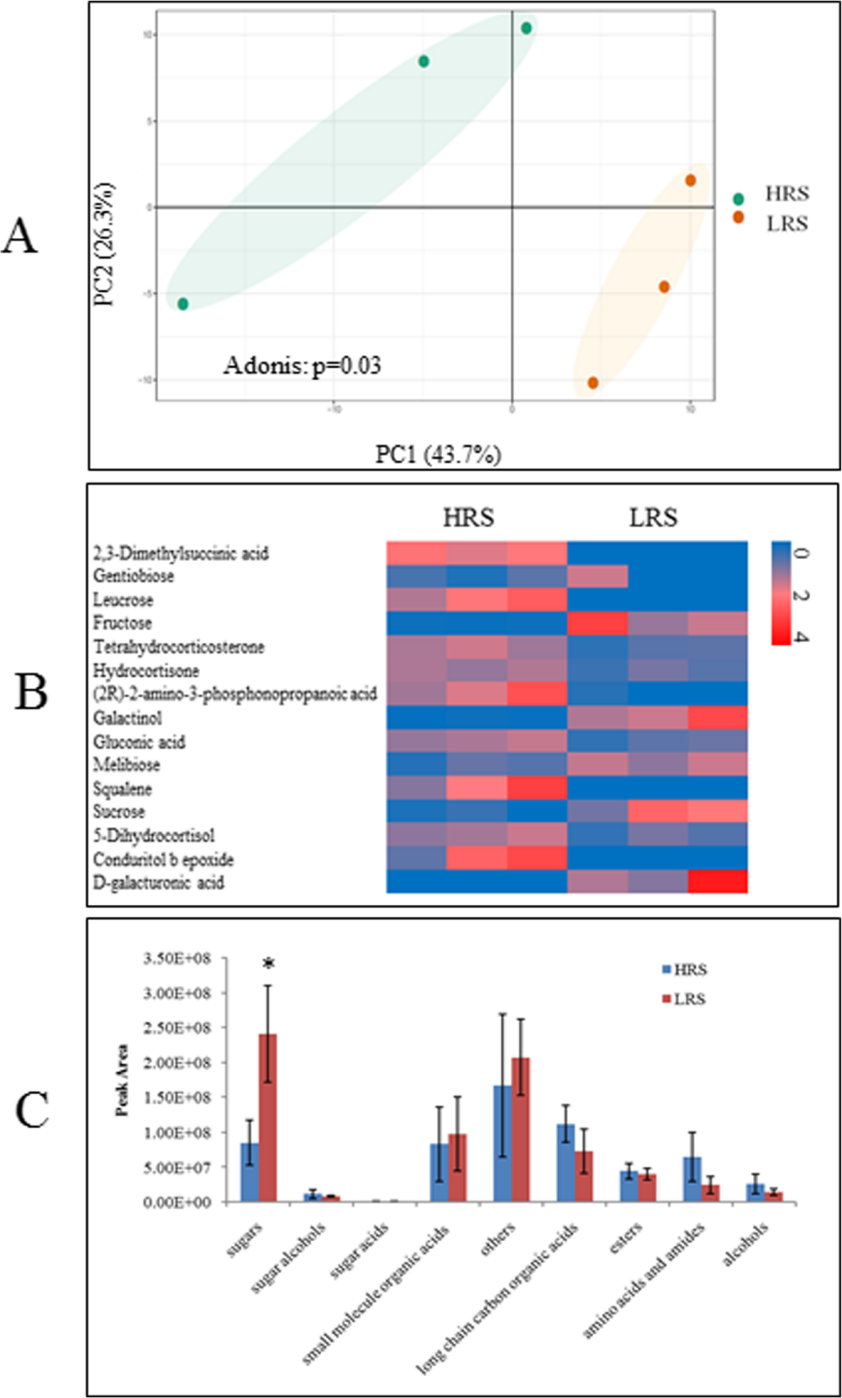
**a** Principal component analysis of rhizosphere soil small molecular profiles. **b** Heatmap analysis of changes in abundance of representative compounds that were significantly different in abundance between two groups. **c** Abundance (cumulative peak area) of compound categories. Each bar represents the average of three replicates. Asterisk indicated the significant difference between two groups

In order to establish a mechanistic explanation about the effects of pathogen abundance on the rhizosphere soil microbiome, rhizosphere soil metabolites of high and low RS abundance were collected and analyzed by gas chromatography-time of flight mass spectrometry (GC-TOF-MS). Principal component analysis (PCA) based on all of the 694 detected peaks revealed the clearly different patterns of rhizosphere soil metabolites between LRS and HRS treatment (AMOVA, *p* < 0.05, Fig. 6a). According to the structure of the molecules, 249 of the 694 detected peaks were divided into 8 categories, including sugar alcohols (8), sugars (38 compounds), sugar acid (4), low molecular weight organic acids (31), long-chain organic acids (37), esters (13), amides (40), amino acids, alcohols (22), or others (56). Differential analysis indicated that 16 compounds of them showed significant differences (t-test, p < 0.05) between the two groups (Fig. 6b). When evaluated them upon the above classification level, sugars and small molecule organic acids were higher in LRS treatment, but only sugars showed a significant difference (t-test, p < 0.05) (Fig. 6c). Overall, rhizosphere soil from LRS group harbored a significantly more abundance of sugars, especially fructose, sucrose and melibiose.

Mantel tests showed a positive and significant correlation between rhizosphere soil metabolites and bacterial phylogeny (Person: *r* = 0.44, *p* = 0.006). Partial CCA-based variation partitioning analysis (VPA) demonstrated that rhizosphere soil metabolites explained 17.2% of variance.

## Discussion

Here, we report the variations of the tomato rhizosphere microbiome in association with high and low abundance of pathogen *R. solanacearum*. Results indicated that pathogen content in the rhizosphere significantly impacted the composition and diversity of bacterial community. The relative abundance of *Bacteroidetes* was significantly dominated by the enrichment of RS within *Proteobacteria*. Similar trend occurred when the tomato rhizosphere was infected by *P. parasitica* [21]. Generally, the low microbial diversity in the rhizosphere soil is beneficial for the pathogen invasion [24]. Indeed, in our study the success of pathogen invasion decreased the microbial diversity as shown by the significant decrease in Shannon, Chao and Faith’s PD indexes at high RS abundance samples, which indicated that pathogen invasion disrupted the host associated microbiome [43]. Principal coordinates analysis showed that the alteration of pathogen abundance altered the composition of bacterial community, and analysis after removing OTU of RS further supported this conclusion. The abundance of several beneficial genera (*Pseudomonas*, *Burkholderia*, and *Bacillus*) was negatively correlated with that of pathogen. The species of these beneficial genera are reported to play important roles in controlling soil-borne disease in disease-suppressive soils by producing antimicrobial secondary metabolites and volatile organic compounds [6, 26, 34, 45].

The correlation analysis of the abundances of beneficial microbes and pathogen has been used to recognize potential anti-pathogen microbes in some publications [9, 28]. However, we cannot consider them as the beneficial bacteria only by simple correlation analysis for almost all of the taxa were relatively decreased due to the higher abundance of the pathogen. Further investigations are required to design effective screening for isolation of anti- *Ralstonia* microbes.

Interactions among rhizosphere microbes are important not only for the stability of the community but also for the host development. Generally, more connections within the community indicate its stability with capacity of restraining pathogen invasion [39, 43]. In this study, the high RS abundance was found to destroy the bacterial connections of tomato rhizosphere. *R. solanacearum* with the highest relative abundance showed the least connections with other microbes. Only one OTU classified as *Kaistobacter* was connected to RS in both HRS and LRS samples. *Kaistobacter* has been reported to be more prevalent in suppressive soils [18, 20, 23], and it is a potential PGPR group because it can produce hormone [19]. Co-occurrence network analysis indicated that the RS invasion affected rhizosphere microbe networks, furthermore, the *Flavisolibacter* with negatively correlated to RS is one important hub bacteria in HRS networks. Both *Kaistobacter* and *Flavisolibacter* can biologically control the bacterial wilt disease caused by *R. solanacearum*, but this hypothesis needs further verification.

The rhizosphere microbiome which showed a direct effect on plant health and resistance to pathogens [27, 33] was mastered by rhizosphere soil metabolites [26, 47]. Root exudates depend on many abiotic and biotic factors, with the former including pathogen invasion, especially colonization. For example, *Fusarium oxysporum* colonization could stimulate the secretion of phenolic acids [46]. Foliar pathogen infection increased the concentration of some amino acids and fatty acids in root exudates [47]. While a little knowledge of rhizosphere soil metabolites impacted by pathogen especially RS was revealed till now. Our study showed that the higher pathogen abundance in tomato rhizosphere significantly altered the soil metabolite profile compared to the lower pathogen abundance samples. Sugars abundant in low RS samples indicated that high sugars were not beneficial for *R. solanacearum* growth. It was likely because sugars, such as fructose and sucrose, were not carbon source for *R. solanacearum* [44]. Indeed, fructose and sucrose were abundant in low RS rhizosphere samples. Amino acids were associated to *R. solanacearum* invasion [15], because they had a higher relative concentration in HRS than LRS rhizosphere soil samples. Several other compounds showed significantly positive or negative correlation with pathogen abundance but further studies should verify their effects on pathogen colonization.

## Conclusion

Interactions between host plants and pathogen in vivo are research hotspots in the field of plant protection. While the interactions happened in rhizosphere become attractive in plant and soil studies. Pathogen invasion even colonization can alter the composition of rhizosphere soil metabolites as well as microbial community, while in turn, the altered metabolites and microbial community can affect the colonization of pathogen [40, 43]. This kind of dynamic cause-and-effect interaction results in a complex system. This study revealed the features of rhizosphere microbiome and metabolites, with the pathogen invasion reducing the diversity and changing relative abundance of some groups. The high abundance of pathogen invasion also reduced the concentration of many sugars in the rhizosphere soil. However, our study did not clarify how microbial groups or metabolites were changed, by pathogen invasion or by host plant? Further studies are needed to better understand the interactions in rhizosphere between pathogen and host plants.

## Methods

### Experiment description and soil sampling

The greenhouse experiment of tomato (“Shi-Ji-Hong-Guan” (production ID: 1880518) is a commercial variety in China purchased from Jiuquan Sanbao Seed CO., LTD, which has not been deposited in any publicly available herbarium) was carried out at the Nanjing Institute of Vegetable Science (31°43′ N, 118°46′ E), Nanjing, Jiangsu province, China, which was a 7-year-monoculture long-term field for studying tomato bacterial wilt. For this study, tomato plants were planted from 22nd, March 2017 to 20th, June 2017, and the rhizosphere samples were collected on 7th June 2017 before the harvest of tomato fruit. In detail, based on visual observations of tomato bacterial wilt symptoms, the roots of healthy and weak tomatoes (9 replicates for each group) from the experiment site with 100% disease incidence previously. Then, all root samples were aseptically transferred to storage bags and maintained on ice prior to transport to the laboratory immediately. The rhizosphere soil samples were obtained according to Fu et al [14]. Briefly, soil loosely adhered to the tomato roots was shaken off and discarded, then the root tissues with their associated rhizosphere soil were cut into 1 cm segments by using a sterile scalpel under aseptic conditions. Sterile water was used to rinse the soil which closely bound to the tomato root segments. For collection of low molecular weight molecules, half of the suspension was taken out for lyophilization. The rest of them were centrifuged at 15,000 g for 20 min and the precipitations were used to extracted DNA.

### Soil genome DNA extraction and *Ralstonia solanacearum* quantification

The rhizospheric soil samples were subsequently extracted the total DNA using the UltraClean Soil DNA Isolation Kits (MoBio Laboratories Inc., Carlsbad, USA) according to the manufacturer’s protocol. Two technical replicates per sample were used to minimize the DNA extraction bias, negative controls were also performed because of uncontrolled DNA polluted [36, 42]. Samples were stored at − 20 °C, and performing polymerase chain reaction was performed with the pooled DNA samples of technical replicates. The NanoDrop ND-2000 spectrophotometer (NanoDrop, ND2000, Thermo Scientific, 111 Wilmington, DE) was used to assess all DNA sample quality based on the 260/280 nm and 260/230 nm absorbance ratios. The concentration of extracted DNA ranged between 40 ng/μl and 60 ng/μl. *Ralstonia solanacearum* abundance was determined by quantitative PCR using primers targeting the *fliC* gene that coding the flagella subunit (forward primer: 5′-GAA CGC CAA CGG TGC GAA CT-3′ and reverse primer: 5′-GGC GGC CTT CAG GGA GGT C-3′) on Applied Biosystems StepOne Plus (Applied Biosystems, CA, USA) [37]. The standard curves generation and qPCR assay were performed as described in our previous publication [49]. Samples were separated into two groups based on the abundance of RS (*Ralstonia solanacearum*): high abundance of RS (HRS) and low abundance of RS (LRS).

### PCR amplification and sequencing

Primers 338F/806R were used to amplified the V3-V4 part of the 16S rRNA gene of the extracted DNA [11]. The PCR amplification with the following amplification cycles: 95 °C for 5 min -- 30 cycles of 94 °C for 30 s + 52 °C for 30 s + 72 °C for 30 s -- 72 °C for 10 min. For amplification, the 25 μL reaction mixtures with 1 μL each primer (10 μM), 5 μL 5 x Q5 GC high enhancer, 2 μL deoxynucleoside triphosphates (dNTPs), 9.75 μL of sterilized ultrapure water, 5 μL 5 x PCR buffer, 0.25 μL 5 U/μl of Q5 polymerase and [8] and 1 μL DNA (20 ng/μL). The amplicon process was performed based on the reported best practices [36, 42]. The bands were excised and purified from 1.2% agarose gels using the MinElute PCR Purification Kit (Qiagen, Germany), and the QiagenQIAquick Gel Extraction kit (Qiagen, Germany). The PCR products were mixed and paired-end sequencing on the Illumina MiSeq sequencing platform at Guangdong Magigene Biotechnology Co., Ltd. China.

### Amplicon sequence processing and analysis

The 16S rRNA gene sequences were processed using QIIME1.9.1 [5], USEARCH 10.0 and in-house scripts. Paired-end Illumina reads were filtered by FastQC and joined by join_paired_ends.py script. Based on the high-confidence 16S representative sequences, an OTU table was generated by USEARCH (−usearch_global and uc2otutab.py scripts, cutoff = 0.97). Another filter step was performed to remove non-bacterial 16S rRNA gene sequences by aligning representative sequences of all OTUs to the Greengenes_13.5 database using PyNAST (align_seqs.py script). The taxonomy of the OTUs was classified with the Greengene_13.5 classifier.

To describe the composition of the rhizosphere community, 9519 sequences was extracted randomly for each sample to calculate *alpha* diversity index, included Shannon, Chao, and Faith’s PD indices, which estimated the *alpha*_diversity.py script by Qiime. A phylogenetic tree was constructed using the script make_phylogeny.py (with default settings – FastTree). Before calculation of *beta* diversity, we first used the CSS method to standardize the OTU profiles by normalize_table.py script [59]. and Bray-Curtis similarity matrices were prepared using the beta_diversity.py script. The Principal coordinate analysis (PCoA) plots were generated from Bray-Curtis similarity matrices created using ggplot2 package in R version 3.4.3. For better evaluation the effect of the RS on rhizosphere bacterial community, we re-draw the PCoA plots excluding the 16S rRNA gene sequence of RS. Furthermore, the phylogenetic molecular ecological network analysis (MENA) [10, 50] was used to explore interaction networks in the two treatments, For MENA analysis, the OTU table was filtered by removing OTUs which relative abundance below 0.001 before uploading to the pipeline. The OTUs which at least in three of all samples were retain, some OTU which have missing value were kept blank and the Pearson correlation coefficient was selected for calculate similarity of OTUs. The correlations of the correlation matrix were filtered with correlation values ≥0.96, *p*-value < 0.05. The global properties of the networks were calculated in MENA pipeline. Gephi (v. 0.92) was used to visualize the networks finally [1].

### Detection and analysis of low molecular weight molecules by GC-MS

Three rhizosphere soil samples from high RS abundance group or low RS abundance group were randomly picked (six in total) for the metabolite analysis. For the extraction of low molecular weight molecules, soil samples were divided into two parts (0.2 g for each) in 2 mL EP tubes and 24 μL of Adonitol (1 mg/mL stock in dH_2_O) were added as an internal standard. One part of the soil samples were homogenized in ball mill for 4 min at 45 Hz in 0.5 mL methanol solution (*V*_methanol_: *V*_H2O_ = 3:1), then ultrasound treated for 5 min (incubated in ice water) for 5 times. The supernatant (0.4 mL) was transferred into a fresh centrifuge tube after centrifuged for 15 min at 10000×g at 4 °C. The residue was extracted with 0.5 mL ethyl acetate using the method mentioned above: then 0.4 mL of ethyl acetate extraction was transferred to the methanol extraction. The other part of the soil sample was extracted by ethyl acetate followed by methanol using the method reported above. Overall, 1.6 mL (0.4 × 4) solution was obtained from one soil sample, and 40 μL were taken and pooled as QC sample, and 1.2 mL were transferred into a fresh 2 mL GC/MS glass vial and dried with nitrogen gas. Then 20 μL methoxyamination hydrochloride (20 mg mL^− 1^ in pyridine) were added to the dried sample and incubated for 30 min at 80 °Cbefore being treated with 30 μL of the BSTFA (bis (trimethylsilyl) trifluoroacetamide) regent (1% TMCS (Trimethylchlorosilane),v/v); finally the mixture was incubated for 1.5 h at 70 °C. The GC-TOF-MS analysis and the raw peaks analysis were performed as the same as reported by Li et al. [22]

For rhizosphere soil metabolome analyses, principal component analysis (PCA) [60] was used to visualize the composition of two samples using the R package vegan. The pathway analysis was displayed by MetaboAnalyst (https://www.metaboanalyst.ca//faces/ModuleView.xhtml).

### Statistical analysis

Differences of alpha-diversity indexes between groups were tested using a nonparametric t-test in R revision 3.4.3. The differences of bacterial community composition and root exudates between the two treatments were tested using PERMANOVA (Permutational multivariate analyses of variance, Adonis, transformed data by Bray-Curtis, permutation = 999) and AMOVA (analysis of molecular variance). To determine the percent change in root exudates, we used t-tests for all compounds of rhizosphere soil metabolome with relative abundances to measure the significant difference in these abundances between the two samples. The *P*-values were corrected by the Benjamini-Hochberg FDR procedure for multiple comparisons [2]. All plots were created using R except the enrichment analysis of metabolic pathways plot.

## Supplementary information

**Additional file 1: Figure S1.** Principal coordinates analysis (PCoA) with Bray-Curtis dissimilarity of the rhizosphere bacterial communities using the whole OTU table excluding the OTUs belonging to *Ralstonia solanacearum*.

**Additional file 2: Table S1.** The characters of co-occurrence networks of two groups with different abundance of RS.

## Data Availability

Raw sequences data were deposited in the NCBI Sequence Read Archive (SRA) database under accession number SRP246782 (https://www.ncbi.nlm.nih.gov/sra/?term=SRP246782).

## Abbreviations

OTU: Operational taxonomic units
GC-MS: Gas Chromatography - Mass Spectrometry
RS: *Ralstonia solanacearum*
LRS: lower RS abundance group
HRS: higher RS abundance group
PCoA: Principal coordinates analysis
