# Supplementary figures and images for: High abundance of Ralstonia solanacearum changed tomato rhizosphere microbiome and metabolome
Source: BMC Plant Biol. 2020 Apr 15;20:166. doi: 10.1186/s12870-020-02365-9 (PMC7160980; doi:10.1186/s12870-020-02365-9)

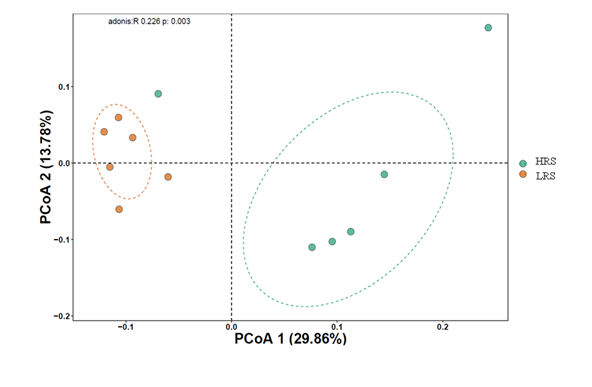

Supplement: Supplementary file 1 — Additional file 1: Figure S1. Principal coordinates analysis (PCoA) with Bray-Curtis dissimilarity of the rhizosphere bacterial communities using the whole OTU table excluding the OTUs belonging to Ralstonia solanacearum. [file 12870_2020_2365_MOESM1_ESM.tif]
