# Supplementary material for: High abundance of Ralstonia solanacearum changed tomato rhizosphere microbiome and metabolome
Source: BMC Plant Biol. 2020 Apr 15;20:166. doi: 10.1186/s12870-020-02365-9 (PMC7160980; doi:10.1186/s12870-020-02365-9)
Supplement: Supplementary file 2 — Additional file 2: Table S1. The characters of co-occurrence networks of two groups with different abundance of RS. [file 12870_2020_2365_MOESM2_ESM.docx]

Supplementary Table 1

The characters of co-occurrence networks of two groups with different abundance of RS

| Network Indexes | HRS | LRS |
| --- | --- | --- |
| Total nodes | 197 | 310 |
| Total links | 666 | 2153 |
| R square of power-law | 0.52 | 0.565 |
| Average degree | 6.761 | 13.89 |
| Average clustering coefficient | 0.491 | 0.189 |
| Average path distance | 5.639 | 3.222 |
| Centralization of degree | 0.089 | 0.235 |
| Centralization of betweenness | 0.2 | 0.085 |
| Maximal stress centrality | 569825 | 129935 |
| Connectedness | 0.463 | 0.84 |

Connections were drawn between nodes that were significantly (P < 0.01; Spearman's rank correlation test) and highly (Spearman's r > 0.96) correlated.
